# Evaluating Somfit’s pulse arterial tonometry for detection of obstructive sleep apnoea

**DOI:** 10.1007/s41105-024-00559-4

**Published:** 2024-11-27

**Authors:** Marcus McMahon, Jeremy Goldin, Elizabeth Susan Kealy, Darrel Joseph Wicks, Eugene Zilberg, Warwick Freeman, Behzad Aliahmad

**Affiliations:** 1 Department of Respiratory and Sleep Medicine, Epworth Hospital, Richmond, Victoria, Australia; 2 Department of Respiratory and Sleep Medicine, Austin Health, Heidelberg, Victoria, Australia; 3 Department of Respiratory and Sleep Medicine, Royal Melbourne Hospital, Parkvile, Victoria, Australia; 4 Sleepmetrics Pty Ltd, Heidelberg, Victoria Australia; 5 Sleep Disorders Unit, Epworth Hospital, Richmond, Victoria, Australia; 6 Medical Innovations, Compumedics Limited, Abbotsford, Victoria, Australia

**Keywords:** Home sleep apnoea testing, Somfit, Polysomnography, Pulse arterial tonometry, Obstructive sleep apnoea

## Abstract

This study evaluates the diagnostic accuracy of Somfit against polysomnography (PSG) for detecting obstructive sleep apnoea (OSA). Somfit is a wearable home-sleep monitoring device attached to the forehead, combining pulse arterial tonometry, oximetry, and actigraphy with sleep staging, arousals, and total sleep time (TST) derived from frontal neurological signals. Ninety-two participants suspected of having OSA were assessed using Somfit and simultaneous overnight PSG recordings at three Australian sites. Each PSG study was manually scored by three independent scorers. The reported statistics include standard measures of agreement between Somfit’s TST, Oxygen-Saturation Index (ODI), Apnoea–Hypopnea Index (AHI), and the average of those metrics from the three PSG scorers. The overall inter-scorer agreement was 76% (kappa = 0.772). TST, ODI, and AHI from Somfit were highly correlated with similar metrics from PSG (all r > 0.84, *p* < 0.001). Sensitivity, specificity, and accuracy were 90.5%, 75.0%, and 89.1% respectively, with a diagnostic odds ratio (DOR) of 28.5 for AHI ≥ 5. For AHI ≥ 15, sensitivity, specificity, and accuracy were 78.0%, 73.8%, and 76.1%, respectively, with a DOR of 9.99. For AHI ≥ 30, sensitivity, specificity, accuracy, and DOR were 72.4%, 90.5%, 84.8%, and 24.9, respectively. The area under the curve (AUC) at different PSG AHI cut-offs ranged between 0.86 and 0.93. Coupled with oximetry and EEG-based derivation of TST, Somfit’s performance is comparable to PSG in detecting OSA severity.

## Introduction

Obstructive sleep apnoea (OSA) is the most prevalent type of sleep apnoea [1] that occurs when the muscles in the throat relax during the sleep, obstructing the passage of air to the lungs. It is a health condition characterized by a combination of shallow, restricted breaths that last for 10 s or longer, which are associated with arousal or a drop in oxygen saturation (i.e. hypopnea), and/or a complete collapse of the upper airway during the sleep (i.e. apnoea). The syndrome is linked with a sense of tiredness during the day or persistent fatigue [2]. Untreated, it can lead to poor glucose control [3], hypertension [4], stroke and other cardiovascular disorders [5], impaired cognitive functioning [6], erectile dysfunction [7], and even mortality [8].

Currently, polysomnography (PSG) conducted in a laboratory setting is considered the gold standard for diagnosing OSA. However, given the high prevalence of this condition, the financial implications of using PSG to test all individuals suspected of having OSA are substantial. In addition, PSG setup requires that patients stay overnight at the clinic with multiple electrodes/sensors attached to their various parts of the body and supervised by a sleep technician throughout the night. However, this setup can be extremely uncomfortable for some patients making it difficult for them to sleep naturally in their habitual position [9]. This may compromise the correct assessment of OSA [9]. It often requires a long waiting time due to limited facilities and the growing demand for sleep studies [10, 11].

Advancements in home sleep apnoea test (HSAT) using wearable technology has emerged, with peripheral arterial tomography (PAT) being introduced nearly two decades ago. This technology has been pivotal in identifying sleep-disordered breathing, aiming to simplify, and reduce the costs associated with traditional full PSG. Using this, the variations in arterial pulse volume caused by arterial vasoconstriction and vasodilation can be quantified. A reduction in arterial volume typically signifies heightened sympathetic nervous system activity, which is linked to the end state of respiratory event. Such an occurrence manifests as a marked diminution in the pulse, a signal, which, when combined with an increased heart rate and/or a drop in oxygen saturation, can be indicative of a respiratory disturbance.

Several, PAT-based HSAT devices such as the NightOwl (Ectosense, Belgium) and WatchPAT (Zoll Itamar, USA) have been cleared by the U.S Food and Drug Administration (FDA) as a viable and sometimes preferable alternative to in-lab PSG, for home-based sleep apnoea testing. Such devices utilize PAT, oximetry, and actigraphy to automatically score respiratory events and apnoea–hypopnea index (AHI). While such HSAT devices are more cost-effective, require fewer electrodes compared to PSG, and allow patients to complete the test in the comfort of their own home, there have been some concerns over the diagnostic limitations that could impair the accuracy of scoring sleep apnoea severity due to lack of electroencephalography (EEG) to score true sleep–wake stage, limiting their ability to measure true total sleep time (TST), arousals, and sleep stages [12–14].

**Fig. 1:**
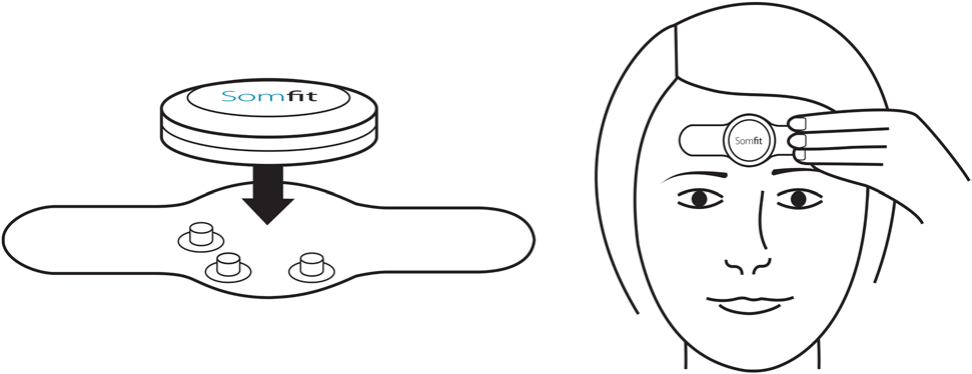
The Somfit device

This paper evaluates the performance of a new HSAT device, the “Somfit”, developed by Compumedics Ltd., Melbourne, Australia (Fig. 1) to address the above limitations. The performance of Somfit’s automatic sleep staging has been studied elsewhere [15] using a large cohort of more than 100 subjects with mixed severity of OSA. It has shown the overall percent agreement of 76.13%, 95% CI [75.27, 76.98] across five sleep stages of N1, N2, N3, REM and Wake, compared to human scoring. In term of Sleep/Wake scoring performance which has direct impact on accuracy of AHI estimation; Somfit has shown good percent agreement of 92.54%, 95% CI [90.55, 94.53] for the normal subjects, 89.91%, 95% CI [88.41, 91.42] for mild OSA, 88.07%, 95% CI [86.14, 89.99] for moderate OSA and 86.83%, 95% CI [85.25, 88.41] for severe cases compared to human scorers.

This paper investigates whether the pulse arterial tonometry-based AHI (pAHI) measured by the Somfit device aligns with the AHI obtained from in-laboratory PSG studies, as the primary objective. Additionally, it examines the agreement between Somfit and PSG in terms of Oxygen Desaturation Index (ODI) and TST, which are the secondary outcome variables influencing diagnosis of OSA.

## Somfit device description

“Somfit” is a new miniaturized level 3 HSAT based on pulse arterial tonometry which has been developed by Compumedics Ltd, Australia, and is attached to the patients’ forehead. It provides frontal neurological signals, representing true EEG, EOG, and EMG for accurate scoring of sleep stages, TST, and arousals. Additionally, it provides pulse arterial tonometry, pulse oximetry, PPG-driven heart rate (HR), PPG-driven respiration rate, temperature, snoring, and accelerometry-derived head positions, angle, tilt, and motion. Essentially, it is a HSAT device, enhanced by the addition of true frontal neurological signals for EEG-based sleep staging which has received FDA clearance for its intended use.

## Data collection

The study originally enrolled 110 participants and involved simultaneous recording of full overnight PSG and Somfit data collected at three locations in Australia, i.e. Sleep Unit, Epworth Hospital, Camberwell Victoria (40 participants), SleepMetrics, Heidelberg Victoria (35 participants) and Appleton Institute Central Queensland University, Wayville South Australia (35 participants).

The participants were selected from a pool of patients who either had a prior diagnosis of OSA or were suspected of having sleep disordered breathing with high pre-test probability of sleep apnoea. Based on the AASM guidelines on follow-up PSG and HSAT in adult patients with OSA, only OSA patients who experienced ongoing fatigue and/or clinically significant weight change of more than 10% [16], despite adhering to the treatment, received a referral for this clinical trial study.

Other criteria for patient exclusion included:

- being less than 18 years of age
- being unable or unwilling to provide consent.
- requiring nursing attendance and/or OSA treatment/ CPAP therapy.
- having any history of skin reaction or allergy to tapes and electrode gels.
- not being on an alpha blocker or any medication known to induce peripheral vasoconstriction and decrease pulse wave amplitude.
- not having history of chronic obstructive pulmonary disease (COPD), atrial fibrillation (AF) and/or congestive heart failure (CHF).

At all three sites, the candidates arrived at the venue post-dinner in the late evening (i.e., 8:30 pm). They were briefed about the study procedure and the test setup by a sleep technologist. They were then asked to read through the patient information and sign a consent form on a voluntary basis. For those subjects who agreed to participate, a sleep specialist set them up according to the instructions and they were free to go to sleep at their usual bedtime. Simultaneous PSG and Somfit data were collected overnight under the supervision of a sleep technologist to rectify any issues that might arise with the devices during the study.

Out of 110 participants, 18 were excluded for the following reasons, leaving a total of 92 subjects for the analysis:

- One subject opted out of the study and left early due to kidney stone.
- Two Somfit recordings ended early due to communication and app shutdown by the subject.
- One Somfit recording had high impedance and frequent electrode disconnections that was not rectified by the attending sleep technologist.
- Four PSG recordings had poor oximetry that underestimated desaturations.
- Six Somfit analysis failed because of noisy PAT signal.
- Four Somfit recordings exhibited poor quality SpO_2_ due to poor electrode positioning.

This multi-site clinical trial was approved by Bellberry HREC (Bellberry Limited Eastwood South Australia, Australia), Protocol No. 2022–10-1133 and was listed on ClinicalTrias.gov (ID: NCT05647746.)

## Data analysis

PSG data were scored according to AASM guidelines by three independent sleep scientists who were blinded to the subject’s identity and questionnaire data. ODI 3% (i.e. oxygen desaturation of 3% or more), TST and AHI values produced by the scorers were averaged and used in the statistical analyses as the ground truth data. All data from Somfit related to study objectives including sleep staging to calculate TST and identify desaturation events as well as the pAHI were generated by the automatic algorithms of the Somfit device without any manual intervention.

The agreement between the gold standard PSG and the Somfit for TST, ODI and AHI was examined using correlation analysis based on Pearson’s method. A simple linear regression was performed to evaluate the relationship between PSG and Somfit for all the three parameters. Bland–Altman plots were used to assess the mean difference and limits of agreement between PSG and Somfit as well as visualizing any systematic and/or proportional bias in the differences and possible outliers. The ability of Somfit pAHI to discriminate OSA severity was evaluated at three cut-off points (≥ 5, ≥ 15, and ≥ 30) by calculating the area under the curve (AUC) using receiver operating characteristic (ROC) analysis.

## Results

**Fig. 2:**
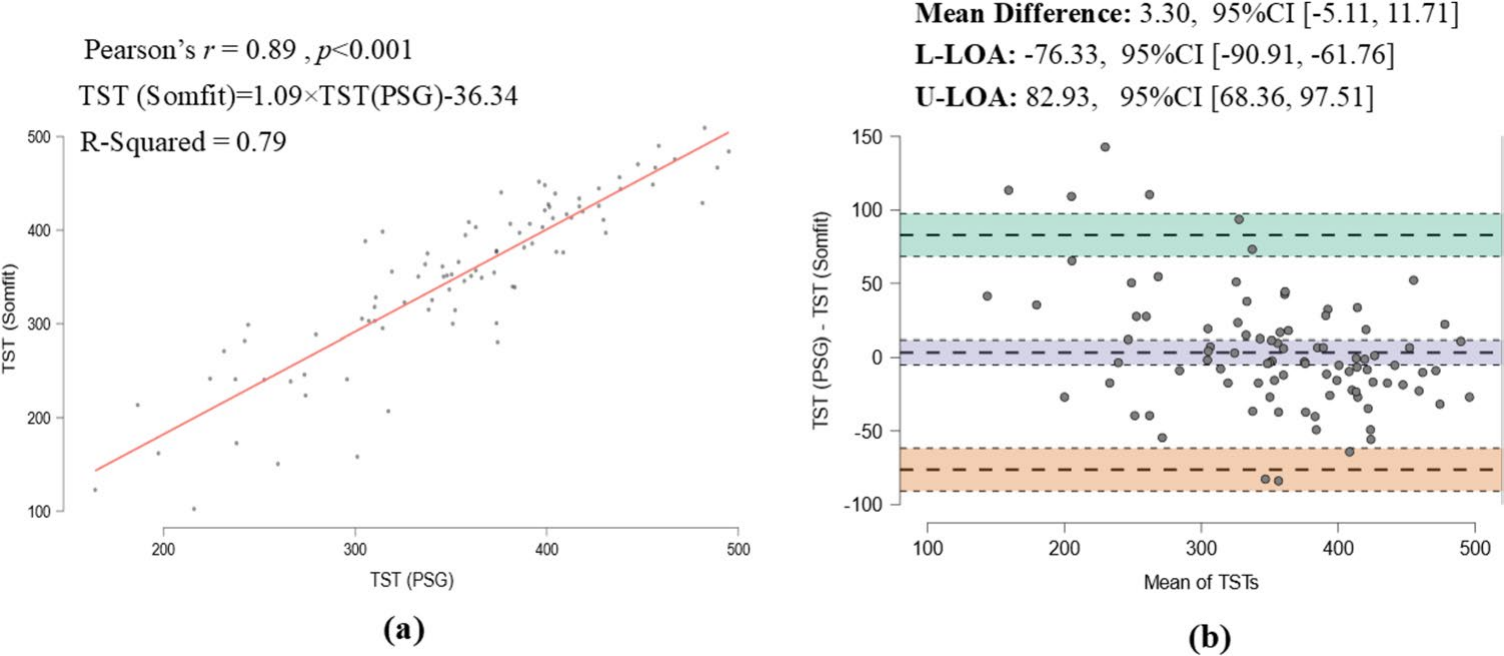
**a** Correlation and **b** agreement between PSG and Somfit TSTs

Table 1 shows the baseline characteristics of the participants split by OSA severity. Of the total cohort (*n* = 92), the overall mean (SD, [Min Max]) age was 55 (12.13, [19 80]) years with nearly 54% of the participants being men. The mean BMI was 30.5 ± 6.89 and mean AHI was 24.5 ± 21.44. Based on the standard AHI cut-offs (i.e. 5, 15 and 30), 8 participants were identified as normal (having no OSA, mean AHI = 2.04 ± 1.52) and 34 with mild, 21 with moderate and 29 with severe OSA. The overall mean (SD, [Min Max]) PSG ODI and AHI were 20.0 (19.27, [0.17 89.3]) and 24.5 (21.44, [0.16 90.6]) events/h, respectively. The inter-rater reliability (Table 2) for scoring OSA severity was calculated using percentage agreement and Fleiss’ Kappa coefficient, resulting in values of 76% and 0.772, respectively. The mean TST reported by PSG was 355 ± 72.3 min and Somfit calculated mean TST of 352 ± 88.6. Examining the Bland–Altman plot (Fig. 2) comparing PSG to Somfit TST showed a systemic bias of close to 0 (i.e. 3.3 min, *95% CI* [-5.11, 11.71]) indicating a good agreement with majority of the data points fell within the two ± 1.96 standard deviations (SD) of the mean PSG [i.e. limits of the agreement (LOA)]. The results of Pearson’s correlation coefficient analysis revealed that Somfit TST was highly correlated with TSTs from the PSG (Pearson’s r = 0.89, *p* < 0.001).

**Table 1:** Descriptive statistics: demographic Info

	OSA Severity	*N*	Mean	SD	Minimum	Maximum
Gender (F/M)	No OSA	8/0				
	Mild	15/19				
	Moderate	11/10				
	Severe	9/20				
	Overall	43/49				
Age	No OSA	8	41.20	15.73	19	61
	Mild	34	57.79	9.65	37	80
	Moderate	21	59.48	11.05	33	75
	Severe	29	53.04	10.65	32	74
	Overall	92	55.0	12.13	19	80
BMI (Kg/m2)	No OSA	8	29.87	11.16	18.900	58.27
	Mild	34	28.15	4.61	20.50	41.00
	Moderate	21	30.84	6.04	21.60	41.00
	Severe	29	33.37	7.20	23.30	55.40
	Overall	92	30.5	6.89	18.90	58.3
ODI 3% (PSG)	No OSA	8	1.15	0.78	0.17	2.6
	Mild	34	7.79	4.90	0.57	28.03
	Moderate	21	16.50	5.27	6.83	26.69
	Severe	29	42.06	19.05	15.33	89.33
	Overall	92	20.00	19.27	0.17	89.33
AHI (PSG)	No OSA	8	2.04	1.52	0.167	4.93
	Mild	34	10.09	2.55	5.52	14.61
	Moderate	21	22.06	3.78	15.10	29.91
	Severe	29	52.78	16.98	33.02	90.60
	Overall	92	24.5	21.44	0.16	90.6

**Table 2:** Inter-scorer reliability

	3 Scorers
Subjects	92
Agreement %	76
Kappa	0.772
Z	21.1
p-value	< .001

**Fig. 3:**
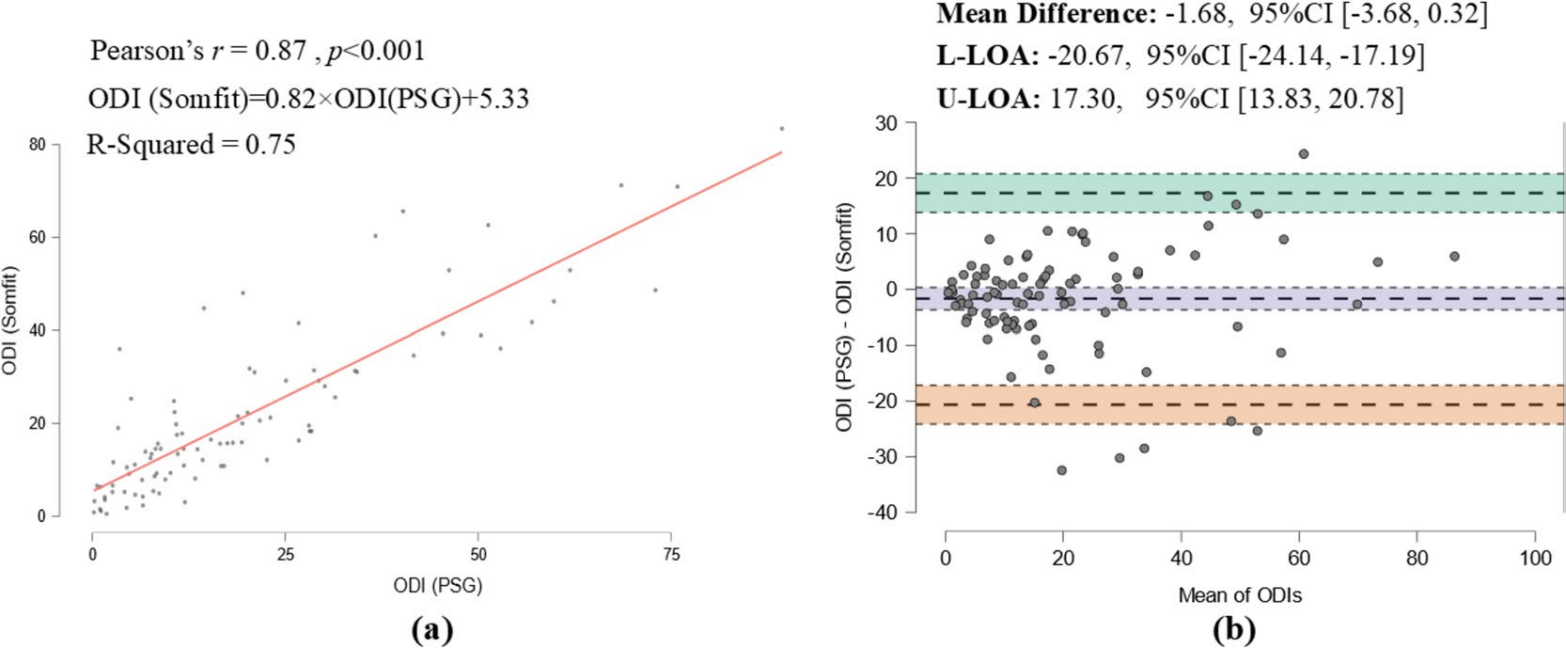
**a** Correlation and **b** agreement between PSG and Somfit ODIs

**Fig. 4:**
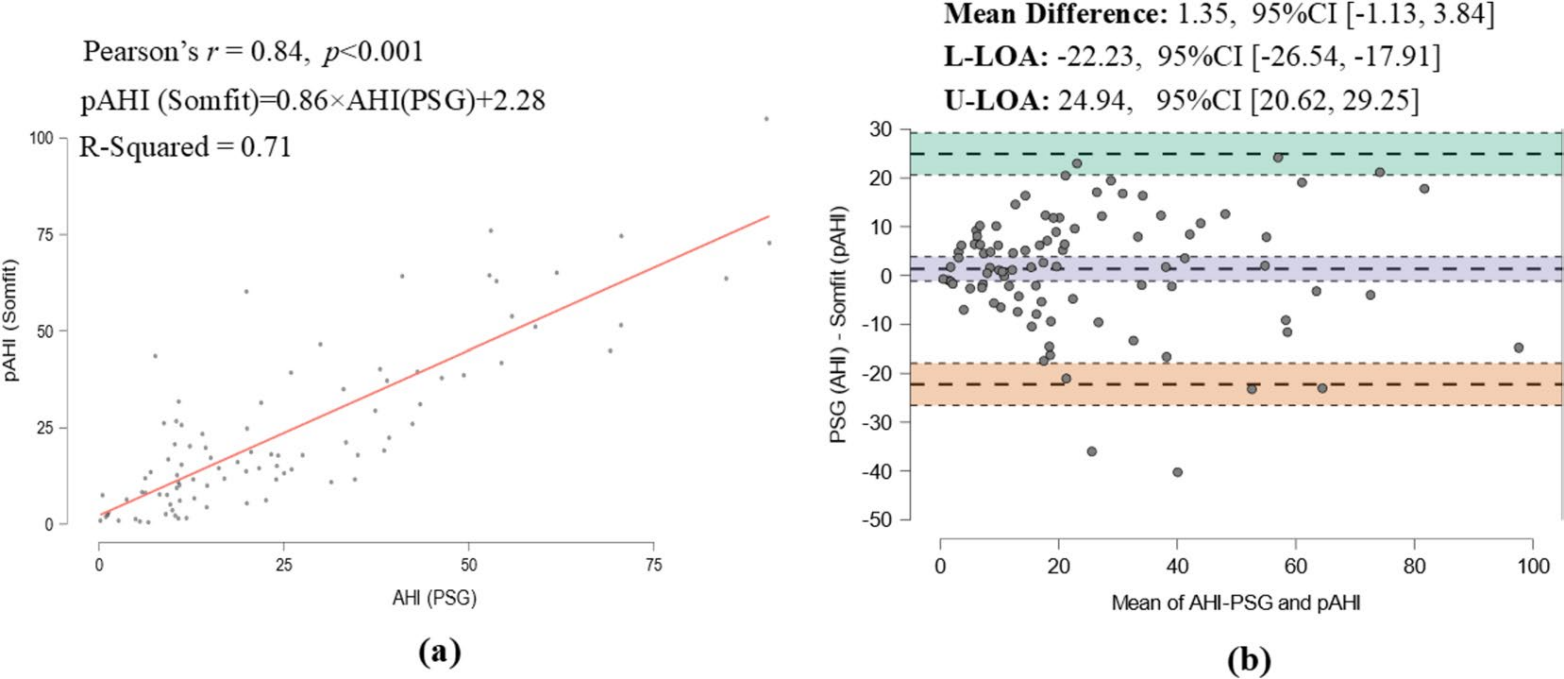
**a** Correlation and **b** agreement between PSG AHI and Somfit pAHI

The mean PSG ODI was 20 ± 19.2 events/h which was in close agreement with the mean ODI of 21.7 ± 18.2 reported by the Somfit, resulting into the mean difference of -1.7 events/h, *95% CI* [-3.68, 0.32]. The Bland–Altman plot (Fig. 3) shows the Somfit ODI corresponded closely to PSG with no visible proportional bias indicating Somfit and PSG agreed equally through the range of measured ODI’s. The data also showed a significant correlation between the ODIs from the two devices (r = 0.87, *p* < 0.001). Similarly, a strong correlation (r = 0.84, *p* < 0.001) was found between pAHI and AHI and a good agreement according to the Bland–Altman plot in Fig. 4, showing the mean difference of 1.35, *95% CI* [− 1.13, 3.84].

**Fig. 5:**
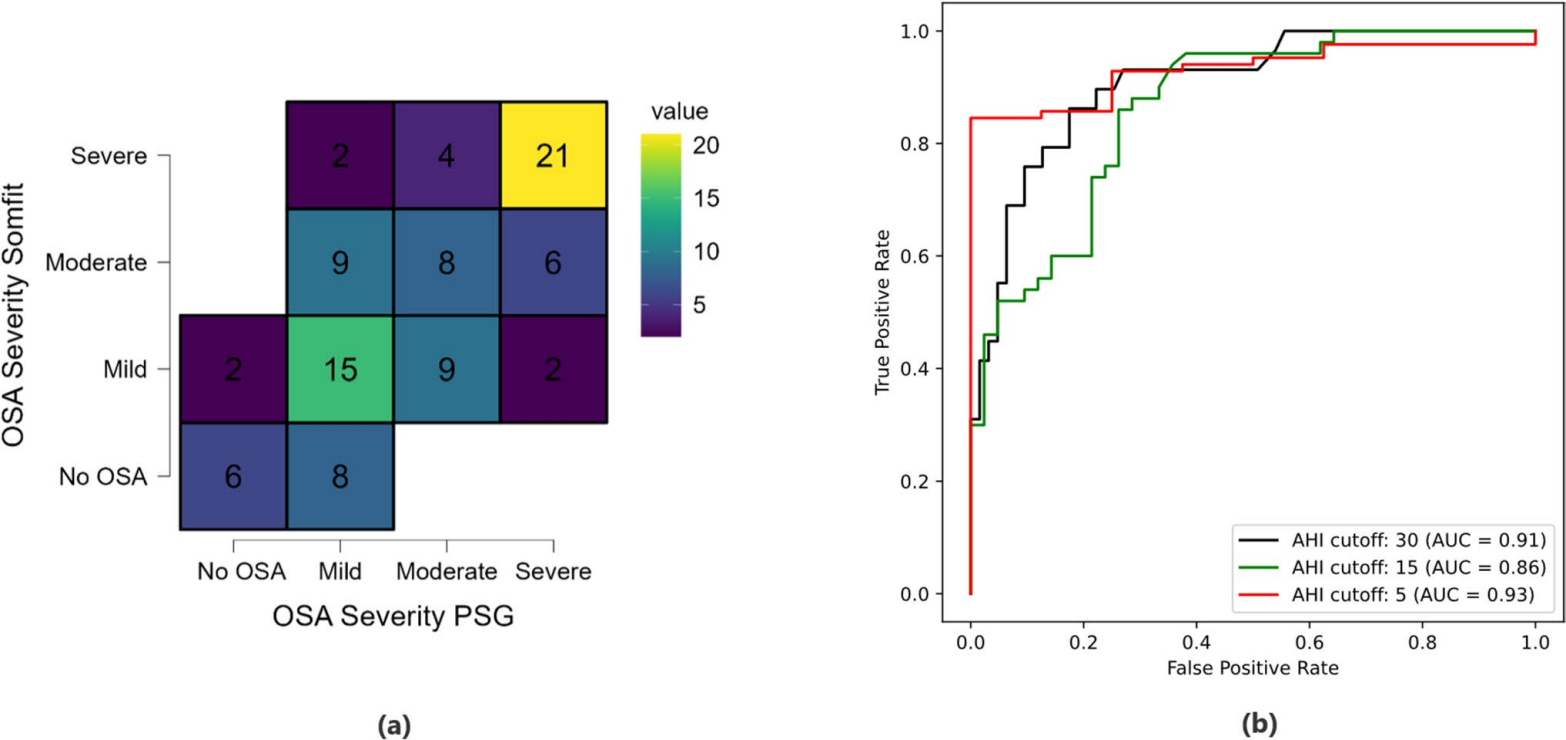
a) Confusion matrix heat map for different categories of OSA severity (i.e. no OSA, mild, moderate, and severe), b) receiver operating characteristic (ROC) curves for OSA severity prediction at AHI thresholds of 5, 15, and 30 events/h with corresponding area under the curve (AUC) values

Figure 5a presents the distribution of PSG AHI (i.e. actual) and Somfit pAHI (i.e. predicted) across the four standard OSA categories of no OSA ( $AHI<5$), mild ($5\le AHI<15$), moderate ($15\le AHI<30$), and severe ($AHI\ge 30$) with the number of predictions shown for each category. The heatmap reveals lighter colours on the diagonal elements of the confusion matrix compared to other elements, indicating a higher proportion of correct predictions.

Table 3 reports the computed sensitivity, specificity, accuracy, and Diagnostic Odd Ratio (DOR) and positive (LR +) and negative (LR-) likelihood ratios at AHI thresholds of 5, 15, and 30 events/h. The sensitivity (%) [95%CI], specificity (%) [95%CI] and accuracy (%) values for AHI ≥ 5 were 90.5 [82.1 95.8], 75.0 [34.49 96.8] and 89.1 respectively. The DOR for this category was found to be 28.5 [4.9 165.3]. For moderate to severe OSA (i.e. AHI ≥ 15) the sensitivity, specificity and accuracy values were 78.0 [64.0 88.5], 73.8 [58.0 86.1] and 76.1 with DOR of 9.99 [3.8 26.0]. The test for severe cases (i.e. AHI ≥ 30) resulted in the sensitivity, specificity and accuracy and DOR of 72.4 [52.8 87.3], 90.5 [80.4 96.4], 84.8 and 24.9 [7.7 80.4] respectively. The ROC curves for discriminating OSA severity (Fig. 5b) demonstrated a good AUC of 0.86 for the AHI cut-off of 15 which was the lowest compared to the AHI thresholds of 5 (AUC: 0.93) and 30 (AUC: 0.91).

**Table 3:** Analysis of diagnostic test accuracy at different AHI thresholds

	Somfit vs PSG (Human score)					
PSG AHI	Sensitivity (%) [95% CI]	Specificity (%) [95% CI]	Accuracy (%)	DOR [95% CI]	LR +	LR-
AHI ≥ 5	90.5 [82.1 95.8]	75.0 [34.9 96.8]	89.1	28.5 [4.9 165.3]	3.62	0.127
AHI ≥ 15	78.0 [64.0 88.5]	73.8 [58.0 86.1]	76.1	9.99 [3.8 26.0]	2.98	0.29
AHI ≥ 30	72.4 [52.8 87.3]	90.5 [80.4 96.4]	84.8	24.9 [7.7 80.4]	7.60	0.30

*AHI* apnoea-hypopnea index, *DOR*  diagnostic odds ratio, LR + and LR–  positive and negative likelihood ratios

## Discussion

This study has investigated the performance of Somfit’s pulse arterial tonometry in identifying the severity of OSA within a sizable adult population, with large prevalence of OSA, using PSG AHI as the gold-standard [17]. The performance of any automatic scoring, when compared to manual methods, should be confined to the context of inter-scorer agreement [18]. AASM inter-scorer reliability program demonstrates a high level of agreement (i.e. 97.4%) among scorers when identifying normal breathing epochs (i.e. no events). However, the agreement significantly reduces when scoring hypopnea (65.4%) and obstructive apnoea (53.4%) with most disagreements in OSA severity occurring in the mild and moderate categories [18]. This is mainly attributed to small variability in the scoring criteria used by different scorers, differences in their levels of experience and complexity of the data. Such factors have led to a notable variance in our manual scores, resulting into moderate interscorer agreement of 76%. Despite such variance, our findings indicate a good agreement with PSG in the automatic scoring of TST, ODI, pAHI and diagnosis of OSA severity by the Somfit device. Somfit performance in discrimination of OSA levels at AHI cut-offs of 5, 15 and 30, as indicated by AUC values, varied from 0.86 to 0.93, performing better at PSG AHI cut-offs of 5 (i.e. mild to severe) and 30 (i.e. Severe) compared to 15 (i.e. moderate to severe). Large DORs of 28.5 [4.9 165.3] and 24.9 [7.7 80.4] for AHI cut-offs of 5 and 30 and relatively lower DOR of 9.99 [3.8 26.0] for moderate to severe groups indicating better discriminatory test performance for the AHI cut-offs of 5 and 30 compared to 15.

In terms of the device failure rate, 7 out of the 13 excluded Somfit studies reported in the ‘Data Collection’ section were primarily due to human error. All HSAT devices can experience human errors when used by patients. However, the simplicity of the Somfit device, which is generally an advantage, may not make it less prone to human errors compared to other devices. Excluding these studies from the total failures results in an overall failure rate of 5.4% (6 out of 110), which is comparable to the failure rate for the WatchPAT device [19]. In the study by Kasai et al.[19], a total of 4 exclusions (out of 128 studies) were reported due to HSAT device failure, with 2 exclusions resulting from data transfer errors and 2 from poor PAT-based device signals, resulting in a failure rate of 3.1% (4/128 × 100). However, contrary to what was mentioned in that paper as another exclusion criterion, the total number of exclusions due to PAT probe not fitting to the patients’ finger, was not provided, and therefore, has not been included in the calculation. Failures of this type do not apply to the Somfit device, as it does not rely on a dedicated finger probe to record the signals.

This study has demonstrated that the Somfit device has performed well compared to similar studies conducted using other HSAT devices. For instance, the study by Ioachimescu et.al [20], involving a large cohort of 500 patients with suspected sleep apnoea and concurrent WatchPAT—PSG recordings revealed that WatchPAT significantly overestimated severity of OSA compared to PSG by an average of 4 events/h when a 3% desaturation threshold for AHI was used (i.e. Mean bias: 4.2, 95%CI [2.8, 5.5]). Conversely, it underestimated disease prevalence and OSA severity by an average of 6 events/h for the desaturation threshold of 4%. The PAT device showed overall accuracy rate of 53.4% (*kappa* = 0.36) for 3% desaturation threshold-based AHI, compared to four PSG diagnostic categories of no OSA, mild, moderate, and severe. Similarly, meta-analysis of 17 studies on 1318 participants who underwent parallel use of PSG and WatchPAT revealed significant discordance with pooled sensitivities of 94.11% and 43.47%, 92.21% and 72.39%, and 74.11% and 87.10% at AHI thresholds of 5, 15 and 30 events/h, respectively [21]. The study also reported a pooled percentage error of 230% between WatchPAT and PSG.

The study has demonstrated the efficacy of Somfit’s pulse arterial tonometry technology, in detecting OSA directly from the forehead. This together with EEG-based estimation of TST, has made the device comparable to PSG. Like every study, this study has a few limitations that could be further improved in the future works. The primary limitation could be the relatively low inter-scorer agreement between PSG scorers at 76% which may have potentially led to an underestimation of the Somfit’s performance. Another weakness of this study was that it was conducted in a single-night in-laboratory setting of the sleep study. Therefore, we could not assess the performance of Somfit when used for unattended home environment. Moreover, this study was performed on a cohort with prior diagnosis of OSA or suspected of having sleep disordered breathing with high pre-test probability of sleep apnoea. Therefore, the ratio of OSA to normal cases were significantly large. This may have led to an increase in the risk of observed false negatives. Further investigation needs to be done on a more balanced population including more normal or patients at low risk of OSA and/or with medical conditions such as COPD, CHF and/or AF.

Based on the results, despite the known limitations, Somfit is recommended as a useful tool for making OSA diagnostic testing more accessible to everyone. As it is worn on the forehead, it enables a true measurement of sleep stages, TST and arousal due to its ability to record frontal neurological signals. Other advantages include being small, lightweight, HSAT device that does not require a dedicated finger probe or a wrist-worn unit. Unlike other HSAT devices that might fail due to reliance on finger oximetry, Somfit could provide an opportunity for patients suffering from chronic poor perfusion in their hands and feet, such as those with peripheral vascular disease, to undergo testing for sleep apnoea.
